# Whole genome sequence data of *Trichoderma yunnanense* strain TM10, a plant growth-promoting fungus and biocontrol agent

**DOI:** 10.1016/j.dib.2025.111283

**Published:** 2025-01-09

**Authors:** Dedat Prismantoro, Kah-Ooi Chua, Kelly Wan-Ee Teo, Rosamond Chan, Thomas Argyarich Jefferson, Nurul Shamsinah Mohd Suhaimi, Muhamad Shakirin Mispan, Mia Miranti, Febri Doni

**Affiliations:** a Department of Biology, Faculty of Mathematics and Natural Sciences, Universitas Padjadjaran, Jatinangor 45363, West Java, Indonesia; b Centre for Research in Biotechnology for Agriculture (CEBAR), Universiti Malaya, Kuala Lumpur 50603, Malaysia; c Institute of Biological Sciences, Faculty of Science, Universiti Malaya, Kuala Lumpur 50603, Malaysia; d Glami Lemi Biotechnology Research Center, Universiti Malaya, Kuala Klawang, Negeri Sembilan 71650, Malaysia; e Department of Global Development, Cornell University, Ithaca, NY 14853, USA

**Keywords:** Genome annotation, MGI sequencing platform, Rice, *Trichoderma yunnanense*, Whole genome sequencing

## Abstract

*Trichoderma yunnanense* strain TM10 was isolated from rhizosphere soil of rice plants cultivated under system of rice intensification (SRI) practises in West Java, Indonesia. It exhibits significant potential as a plant growth promoter and biocontrol agent in rice plants. Although this strain has shown promise in promoting plant growth and suppressing phytopathogens under *in vitro* and *in planta* conditions, there is still a lack of genomic data to elucidate the molecular mechanisms underlying its plant growth-promoting and biocontrol capabilities. This study reports the whole genome sequence of *T. yunnanense* strain TM10. The genome of the fungus was sequenced using the MGI DNBSEQ-G400 high-throughput sequencing platform. The assembled genome of *T. yunnanense* strain TM10 was approximately 36 Mbp in length, comprising 385 contigs with a GC content of 48 % and a sequencing coverage of 43.8×. This genomic data provides a foundation for harnessing the plant growth-promoting and biocontrol potential of this strain. The complete genome sequence has been deposited at the National Center for Biotechnology Information (NCBI) under Bioproject accession number PRJNA1181959, BioSample ID SAMN44575400, and genome accession number JBIYZQ000000000. These data are valuable for further research into the biotechnological potential of this strain and for exploring the molecular mechanisms underlying its plant growth-promoting and biocontrol activities.

Specifications Table

**Table:** 

Subject	Biological Sciences
Specific subject area	Biotechnology, Microbiology, Genomics
Type of data	Table, Figures
Data collection	*T. yunnanense* strain TM10 was isolated from the rhizosphere soil of organic rice plants cultivated under system of rice intensification (SRI) conditions. Genomic DNA was extracted from the pure culture of the fungus using the ZymoBIOMICS™ DNA Miniprep Kit. DNA libraries were prepared using the MGIEasy FS DNA Library Prep Set. Whole genome sequencing was performed using the MGI DNBSEQ-G400 high-throughput sequencing platform. Post-sequencing quality control was conducted using FastQC v0.12.1, followed by trimming and size selection using Trimmomatic v0.39. *De novo* genome assembly was performed with SPAdes v3.11.1. The assembled draft genome was evaluated for quality and completeness using Benchmarking Universal Single-Copy Orthologs (BUSCO) v5.5.0. Gene prediction and annotation of the complete genome were conducted using Funannotate v1.8.15. An ITS gene-based maximum-likelihood phylogenetic analysis was conducted using MEGA XI and a core genes-based phylogenomic analysis was performed using the Funannotate compare annotations tool. The resulting phylogenetic trees were visualized using MEGA XI.
Data source location	City/Town/Region: Tasikmalaya, West Java Country: Indonesia Latitude longitude: 7.127648° S, 108.163728° E
Data accessibility	Data is publicly available at the NCBI repository: Bioproject accession: PRJNA1181959 BioSample accession: SAMN44575400 Genome accession: JBIYZQ000000000 Assembly accession: ASM4583818v1 Direct URLs to data: https://www.ncbi.nlm.nih.gov/bioproject/PRJNA1181959 https://www.ncbi.nlm.nih.gov/biosample/SAMN44575400 https://www.ncbi.nlm.nih.gov/nuccore/JBIYZQ000000000.1 https://www.ncbi.nlm.nih.gov/datasets/genome/GCA_045838185.1/ https://www.ncbi.nlm.nih.gov/Traces/wgs/JBIYZQ01 Supplementary material available at 10.17632/dpyjygt3vj.1
Related research article	D. Prismantoro, T.A. Jefferson, S.I. Akbari, M. Miranti, M.S. Mispan, F. Doni, In vitro inhibition mechanisms of *Trichoderma yunnanense* TM10 against *Pyricularia oryzae* and *Rhizoctonia solani* causing blast and sheath blight diseases in rice (*Oryza sativa* L.), All Life 17 (2024) 2,422,845, 10.1080/26,895,293.2024.2422845

## 1 Value of the Data

• The whole genome sequence of *T. yunnanense* strain TM10 will be a valuable resource for elucidating the molecular mechanisms underlying its potential as a biocontrol agent and plant growth promoter in sustainable rice cultivation. This strain has demonstrated the ability to suppress major rice pathogens, *Pyricularia oryzae* and *Rhizoctonia solani* [1], as well as exhibiting strong potential as a biostimulant by enhancing the growth and physiological characteristics of rice plants [2].
• These data facilitate the identification of genes involved in signalling pathways, secondary metabolite biosynthesis, and enzyme activity that govern interactions between *T. yunnanense* strain TM10, host plants, and pathogens.
• *T. yunnanense* strain TM10 produces cell wall-degrading enzymes (CWDEs) such as chitinase and cellulase, along with plant growth-promoting compounds, including phosphatase, indole-3-acetic acid (IAA), ammonia, and hydrogen cyanide (HCN) [1,2]. Genomic analysis permits reconstruction of the responsible biosynthetic pathways for these compounds, offering deeper insights into their roles.
• The genome data of *T. yunnanense* strain TM10 will be an important resource for researchers in genome mining to identify genes involved in the biosynthesis of metabolites, enzymes, and proteins that contribute to the strain's role as a biostimulant and biocontrol agent in sustainable crop production.

## 2 Background

*Trichoderma* species are well-studied as plant growth-promoting and biocontrol agents for a variety of crops, including rice [3]. These fungi demonstrate considerable potential to enhance agricultural productivity through a range of mechanisms, such as promoting plant growth, inducing abiotic resistance, and suppressing pathogens [4,5]. *T. yunnanense* strain TM10 was isolated from the organic rice fields cultivated using the SRI method in West Java, Indonesia. It has shown promising effects in improving rice growth, including germination, root length, shoot height, and physiological traits such as chlorophyll content [2]. In terms of biocontrol capability, *T. yunnanense* strain TM10 effectively inhibits the growth of *P. oryzae* and *R. solani*, the causative agents of rice blast and sheath blight diseases, respectively. These inhibitory effects are mediated through mechanisms such as competition, mycoparasitism, and antibiosis. Additionally, *T. yunnanense* strain TM10 produces CWDEs, including chitinase and cellulase, which play a crucial role in degrading the pathogen's cell wall, thereby enhancing its biocontrol efficacy [1]. However, there is no genomic data available to fully elucidate the potential of this strain. This study presents the genome data of *T. yunnanense* strain TM10, offering crucial insights into the mechanisms underlying its plant growth-promoting and biocontrol capabilities.

## 3 Data Description

Whole genome sequencing of *T. yunnanense* strain TM10 generated 16,078,243 raw reads. After quality evaluation and filtering, 15,177,402 high-quality reads with an average length of 150 bp were obtained. The assembled genome of strain TM10 is 36,480,673 bp in length, consisting of 385 contigs with an N_50_ contig length of 16,392 bp. The genome has a BUSCO completeness of 97.5 % with reference to the BUSCO gene set for core universal fungal orthologs and an average genome coverage of 43.89×. The genome has a GC content of 48 %. Annotation of the assembly identified 9,348 genes, including 9,116 mRNA and 232 tRNA genes (Table 1).

**Table 1:** Genome features of *T. yunnanense* strain TM10.

Genome features	Values
Genome size (bp)	36,480,673
GC content (%)	48
Coverage	43.89×
N_50_ (bp)	16,392
Number of contigs	385
BUSCO completeness (%)	97.5
Number of predicted genes	9,348
mRNA	9,116
tRNA	232

**Fig. 1:**
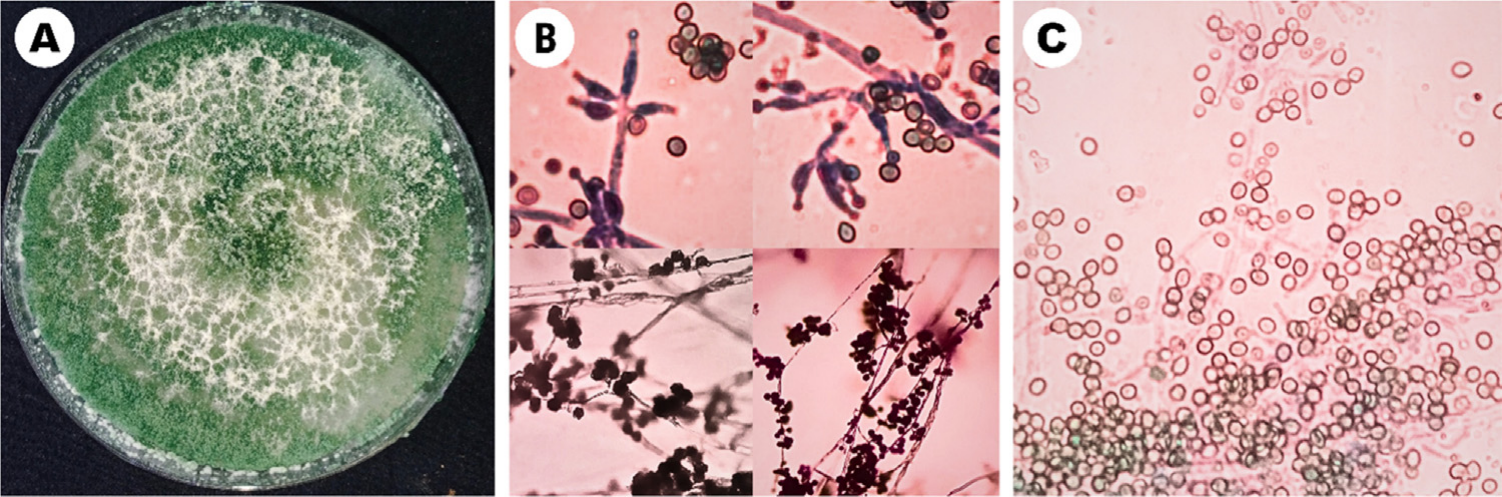
Morphological characteristics of *T. yunnanense* strain TM10, 14 days after inoculation on potato dextrose agar (PDA) medium: (A) Pure culture. (B) Conidiophores and phialides. (C) Conidia (From Prismantoro et al. [1], the journal does not require permission to use the materials).

**Fig. 2:**
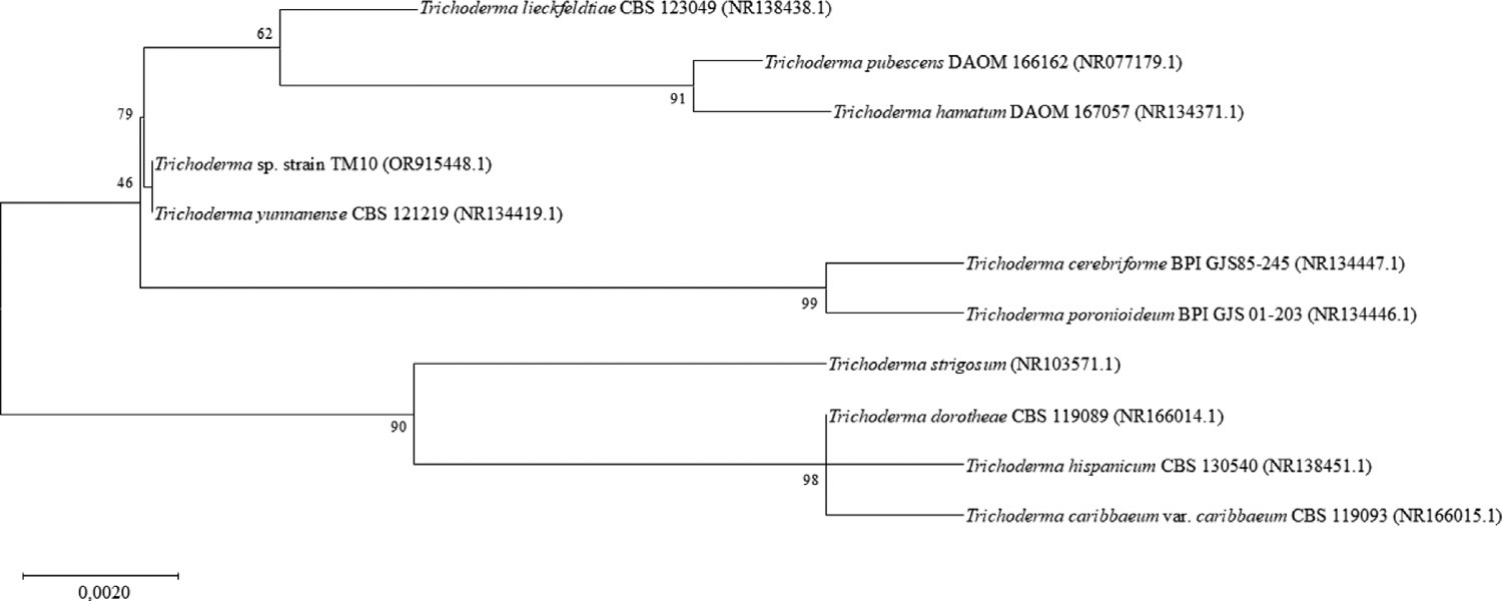
Phylogenetic tree constructed using ITS gene sequences of *T. yunnanense* strain TM10 and closely related *Trichoderma* species. Bootstrap values based on 1,000 replications are indicated at the nodes.

**Fig. 3:**
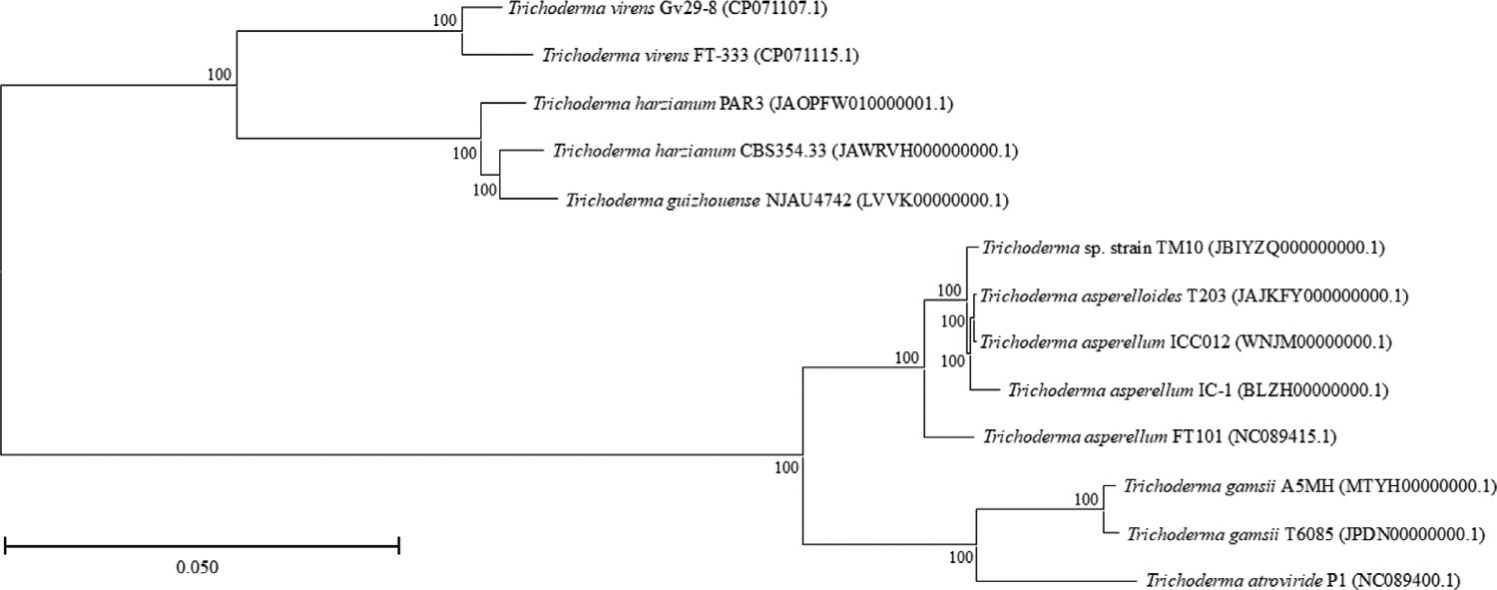
Phylogenomic tree based on core genes sequences of *T. yunnanense* strain TM10 and closely related *Trichoderma* species. Bootstrap values based on 1,000 replications are indicated at the nodes.

The identity of the strain was confirmed through morphological and molecular analyses. After 14 days of incubation, the strain exhibited dark green and white cotton-like mycelium (Fig. 1A). Microscopic examination revealed detailed morphological structures, including conidiophores, phialides (Fig. 1B), and conidia (Fig. 1C). The internal transcribed spacer (ITS) sequence of strain TM10 shared 99 % similarity with *T. yunnanense* strain CBS 121219 based on a BLAST search against the NCBI database. Phylogenetic analysis of the ITS sequence, as shown in Fig. 2, placed strain TM10 in a close cluster with *T. yunnanense* strain CBS 121219, indicating that the strain was most closely related to *T. yunnanense*. However, there is currently no genomic data available for *T. yunnanense*. In subsequent phylogenomic analysis using the core genes shared by all the compared *Trichoderma* genomes, strain TM10 formed an outgroup in a clade that included *T. asperellum* strains ICC012 and IC-1 and *T. asperelloides* strain T203 in the phylogenomic tree. The topology of the tree was supported by a high bootstrap value of 100 %, indicating a confident robustness of the analysis (Fig. 3).

## 4 Experimental Design, Materials and Methods

### 4.1 Isolation of *T. yunnanense* strain TM10

*T. yunnanense* strain TM10 was isolated from the rhizosphere of rice plants cultivated under the SRI method at the SRI Mukti Sadaya Organic Rice Farm in Tasikmalaya, West Java, Indonesia (7.127648° S, 108.163728° E). The fungal strain was cultured on PDA, incubated at 25 ± 2 °C for 7 days. The mycelial mass was harvested and sent to the Saraswanti Genomic Institute in Bogor, Indonesia, for whole genome sequencing.

### 4.2 Morphological and molecular identification

Macroscopic characteristics of the colony (mycelial color and surface texture) and microscopic characteristics (conidiophores, phialides, and conidia) were examined using light microscopy (Olympus CX21, Tokyo, Japan). To confirm the identity of strain TM10, PCR was performed to amplify the ITS region using universal primers ITS1 (5′-TCCGTAGGTGAACCTGCGG-3′) and ITS4 (5′-TCCTCCGCTTATTGATATGC-3′) [6]. The resulting sequence was compared with the NCBI database using the BLASTn program [7]. The ITS sequence of strain TM10 has been deposited in the GenBank database under the accession number OR915448.

### 4.3 DNA extraction and library preparation

DNA extraction of *T. yunnanense* strain TM10 was performed using the ZymoBIOMICS DNA Miniprep Kit (Zymo Research, Irvine, CA, USA), following the manufacturer's instructions. The quality and quantity of the extracted DNA was assessed by 0.8 % agarose gel electrophoresis and the Qubit BR-DNA Assay, respectively. Library preparation was conducted using the MGIEasy FS DNA Library Prep Set (BGI, Shenzhen, Guangdong, China), following the manufacturer's protocol. Genome sequencing was performed using the DNBSEQ-G400 (MGI Tech, Shenzhen, China) flowcell for paired-end sequencing with a read length of 150 bp for each end (PE150).

### 4.4 *De novo* genome assembly

The quality of the raw sequence reads was evaluated using FastQC v0.12.1 [8], followed by preprocessing quality filtering with Trimmomatic v0.39 [9,10]. *De novo* genome assembly was performed using SPAdes v3.15.4 [[11], [12], [13]], and the completeness of the assembled genome was assessed using BUSCO v5.7.1 [14,15]. Gene prediction and annotation were performed using Funannotate v1.8.15 on the Galaxy server v24.1, with default parameters [16].

### 4.5 Phylogenetic analyses

The ITS gene sequences were extracted from the assembled genome and a BLAST search with reference to the NCBI database was done to identify the closely related species [17,18]. The ITS gene sequences of closely related type taxa were obtained from the NCBI database and aligned in MEGA XI using ClustalW. An ITS gene maximum-likelihood (ML) phylogenetic tree was constructed in MEGA XI with 1,000 bootstrap replicates [19]. The whole genome sequences of *Trichoderma* species closely related to strain TM10 were retrieved from the NCBI database for phylogenetic analysis. The information about the reference genomes is shown in Supplementary Table 1. A core gene-based phylogenomic analysis was performed using the Funannotate v1.8.15 compare annotations tool available on the Galaxy server v24.1, and the resulting tree was visualized using MEGA XI [20].

## Limitations

Not applicable.

## Ethics Statement

This work does not involve human subjects or animal subjects. The authors declare that this manuscript is original work and has not been published elsewhere.

## CRediT Author Statement

**Dedat Prismantoro:** Conceptualization, Software, Formal analysis, Investigation, Data curation, Visualization, Writing – review & editing, Writing – original draft. **Kah-Ooi Chua:** Software, Formal analysis, Data curation, Validation, Writing – review & editing. **Kelly Wan-Ee Teo:** Software, Formal analysis, Data curation. **Rosamond Chan:** Software, Formal analysis, Data curation, **Thomas Argyarich Jefferson:** Formal analysis, Data curation. **Nurul Shamsinah Mohd Suhaimi:** Writing – review & editing. **Muhamad Shakirin Mispan:** Supervision, Writing – review & editing. **Mia Miranti:** Supervision, Writing – review & editing. **Febri Doni:** Conceptualization, Methodology, Supervision, Project administration, Funding acquisition, Writing – review & editing.

## Data Availability

NCBITrichoderma yunnanense strain TM10, whole genome shotgun sequencing project (Original data).
